# The analysis of tear meniscus parameters during daily soft contact lens wear using optical coherence tomography

**DOI:** 10.1007/s00417-024-06606-7

**Published:** 2024-08-07

**Authors:** Levent Dogan, Gurcan Dogukan Arslan

**Affiliations:** Department of Ophthalmology, Tatvan State Hospital, Bitlis, Turkey

**Keywords:** Soft contact lens, Ocular discomfort, Tear meniscus, Turbidity, Optic coherence tomography

## Abstract

**Purpose:**

To evaluate tear meniscus parameters in soft contact lens wearers (SCL) using optical coherence tomography (OCT) and ImageJ software.

**Methods:**

This prospective study included 50 soft contact lens wearers (group 1: 25 symptomatic SCL wearers (SCLW), group 2: 25 asymptomatic SCL wearers (ASCW)) and 25 healthy non-CL wearers (group 3 (NCLW)). SCLs were fitted on each eye of CL wearers, and the lower tear meniscus was imaged using OCT before CL insertion, immediately afterward, and reimaged 2, 5 and 10 h after insertion. Tear meniscus parameters, including tear meniscus height (TMH), depth (TMD), turbidity, and percentage area occupied by particles (PAOP) were measured in all groups.

**Results:**

Turbidity and PAOP measurements at baseline in SCLW were significantly higher than in other groups (*p* < 0.05). There was no significant difference between TMH, TMD, turbidity, and PAOP parameters calculated at baseline visit and two hours after SCL insertion in all groups (*p* > 0.05 for 2 comparisons). The symptomatic SCL users had a significant decrease in TMH and TMD in the fifth hour. The turbidity and PAOP measurements of SCLW and ASCW at the fifth and tenth hours were significantly higher than those of NCLW (*p* < 0.05).

**Conclusion:**

TMD and height TMH decrease throughout the day in all participants; however, a significant decrease in these parameters was observed only in symptomatic SCL users at the fifth hour, at the earliest. As the duration of CL wear increases, turbidity and PAOP even in asymptomatic SCL wearers become significantly higher than those in healthy non-CL wearers.

**Key Messages:**

**What Is Known**

• Contact lens wear is associated with an increased risk of dry eye.

• Tear volume decreases gradually during contact lens wear.

**What Is New**

• Tear meniscus turbidity and particle area occupied by particles (PAOP) were higher in symptomatic contact lens wearers and they increase gradually during contact lens wear.

• Tear meniscus turbidity and PAOP may be measures of how well the tear film and meniscus are functioning in contact lens wearers.

## Introduction

Contact lens (CL) wear is a widely used method for correcting refractive errors. An estimated 140 million people worldwide, and 45 million people in the United States wear CLs [1–3]. Although contact lenses provide effective vision, wider visual access, a feeling of normalcy, and reduced image distortions and glare, CL use can cause complications such as dry eye, ocular inflammation, and discomfort [4–8]. A significant number of CL wearers suffer symptoms of dryness, itching, and foreign body sensation, especially at the end of the day while wearing CLs, a condition termed CL discomfort (CLD) in the report of the Tear Film and Ocular Surface Society [3, 9–11]. The sensation of dry eye is the most common symptom and it was reported at a rate of nearly 40% in soft CL wearers [12].

Several biophysical tear film changes, including increased evaporation and decreased tear film stability and volume have been reported in CL wearers [13]. Additionally, tear volume is a prominent parameter in the diagnosis of dry eye [14],and Holly et al. showed that the tear meniscus contains 75%–90% of the aqueous fluid volume [15]. Therefore, previously published studies have evaluated the tear meniscus in CL wearers using video microscopy [16, 17], and it has been stated that this technique has some limitations in practical usage, such as low accuracy and repeatability [18, 19]. Firstly, Le et al. have applied spectral domain optic coherence tomography (SD-OCT) to assess changes in the lower tear meniscus in non-lens wearers and CL wearers [14]. The advantage of SD-OCT is that it can provide detailed tomograms in which the meniscus is seen very clearly, and its ease of acquisition allows for the evaluation of changes in the tear meniscus over time [20]. Turbidity refers to the presence of suspended particles in a fluid, leading to its cloudiness and haziness, which is typically not discernible to the naked eye [21]. Previous studies have used SD-OCT to evaluate tear meniscus volume, height, and depth in CL wearers [22, 23], and Carracedo et al. presented the temporal changes in post-lens tear turbidity following prolonged use of scleral contact lenses [24]. However, no-one appears to have evaluated the turbidity of the tear meniscus in conventional CL wearers, so it remains unclear what changes take place under those circumstances. In this study evaluation of the dimensions of the particles in the tear meniscus using SD-OCT images in CL wearers was also aimed.

## Patients and method

This study used a prospective design, and ethical approval was obtained from the Ethics Committee of Van Training and Research Hospital. The study adhered to the tenets of the Declaration of Helsinki, and written informed consent was obtained from each participant. Each subject completed the ocular surface disease index (OSDI) questionnaire, and then underwent a complete ophthalmic examination, including best-corrected visual acuity measurement, intraocular pressure measurement using a pneumatic tonometer, slit-lamp biomicroscopy, fluorescein staining of the cornea and conjunctiva (graded according to Oxford scheme [25]), tear breakup time (TBUT) testing and the Schirmer I test without anaesthesia. Soft contact lens wearers additionally completed the Contact Lens Dry Eye Questionnaire short form (CLDEQ-8). The participants were called 2 days later for OCT examinations to minimize the non-participant factors that can affect the tear meniscus parameters. Three groups of 25 participants were recruited and grouped according to their history of SCL wear and scores obtained in the CLDEQ-8, which consists of eight questions evaluating discomfort, dryness, blurred vision, and the desire to remove the CLs [26]. Group 1, symptomatic CL wearers (SCLW), was composed of participants with at least six months of soft contact lens experience and with a total score of equal or higher than 12 obtained from the CLDEQ-8. Group 2, asymptomatic CL wearers (ASCW), were adapted soft contact lens wearers with no complaints about the use of CLs (total CLDEQ-8 score < 12). Group 3 consisted of healthy, asymptomatic participants who had never used CLs (noncontact lens wearers (NCLW)). The participants in group 1 and 2 had to be SCL users for at least 6 months before being included in the study and had to wear their SCLs at least 5 days per week and 8 h per day. The participants in groups 2 and 3 did not have any history of dry eye, and their OSDI scores were ≤ 12. Seventy-five right eyes of the 75 participants, 50 soft CL (SCL) wearers and 25 healthy non-CL wearers, were enrolled in the study. The refractive error of participants was limited to ± 4.00 spherical and ± 0.75 cylindrical diopters. The exclusion criteria were extended or continuous CL wear, any active ocular disease (except refractive error) or allergy, any systemic disease, and any use of systemic or topical medicine.

**Fig. 1:**
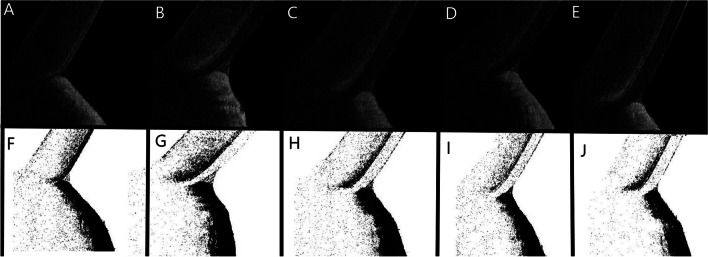
Optical coherence tomography image of the tear meniscus in a participant without SCL (**A**), with immediately after SCL insertion (**B**), 2 h after SCL insertion (**C**), 5 h after SCL insertion (**D**), and 10 h after SCL insertion (**E**). The tear meniscus after software processing to calculate turbidity in a participant without SCL (**F**), with immediately after SCL insertion (**G**), 2 h after SSCL insertion (**H**), 4 h after SCL insertion (**I**), and 10 h after SCL insertion (**J**)

Daily disposable hydrogel lenses (etafilcon A) with base curve 8.5 mm and diameter 14.2 mm were used in this study. The participants in group 1 and 2 were instructed to not wear their CLs for at least 24 h before the visits. Anterior segment and tear meniscus evaluations were performed using the TOPCON 3D MAESTRO 1 (Topcon, Tokyo, Japan) device, which performs 50,000 scans per second and has an axial resolution of 6 µm. Room temperature (24 °C–27 °C) and humidity (40%–50%) were maintained during OCT examination. All images were taken by an experienced examiner in a dimly lit room. To accurately measure tear meniscus parameters, vertical images were taken at the 6 o’clock position of the cornea 2 s after blinking, and this procedure was repeated three times in all examination. Firstly, OCT examination performed without SCLs in all groups. Then, SCLs with refractive-error-specific diopters inserted onto both eyes of CL wearers (SCWL and ASCW) by professional operators and worn for 10 h. There were no statistically significant differences in the CL power between the groups 1 and 2 (*p* = 0.342). OCT examination was repeated immediately after SCL insertion. The consecutive OCT examinations were performed 2, 5 and 10 h after SCL insertion (Fig. 1).

Lower tear meniscus height (TMH) was measured as the distance between the point at which meniscus met the cornea superiorly and eyelid inferiorly using OCT images. The superior border of TMH was SCL in OCT examinations performed with SCL. Tear meniscus depth (TMD) was determined as that of perpendicular distance between the apex of the fornix and TMH. Images obtained using SD-OCT were analysed with ImageJ software (National Institutes of Health, Bethesda, Maryland, USA) to measure tear meniscus turbidity and the percentage area occupied by particles (PAOP). The full tear meniscus was then evaluated. Carracedo et al. presented the change of tear meniscus turbidity with eyedrops instillation and prolonged scleral contact lens wear [24, 27]. In a recent study, Dogan et al. evaluated the tear meniscus parameters in patients with meibomian gland dysfunction using the method outlined Carracedo et al. [28]. In this method, the images were edited and converted into binary, black, and white images in an 8-bit format. A threshold (125, 255) was set in all images to separate the particles from the background. The specific region corresponding to tear meniscus layer was manually selected within the images, and the ‘analyze particles’ command in the software was applied to quantify the number of particles and determine the percentage of area occupied by particles (PAOP). Next, the relationship between the number of particles and total area of the tear meniscus (number of particles per square millimeter) was evaluated. The repeated measurements were robustly performed. The averages of the Image J results obtained from the three OCT images taken for each participant during each visit were used for analysis.

### Statistical analysis

Statistical analysis was performed using SPSS, version 25.0, for Windows (IBM Co, Armonk, NY, USA). All data are presented as mean ± standard deviation. Using the turbidity of the tear meniscus as the primary outcome of the study, a priori sample size calculation based on a significance level of 5%, a power of 80%, and a correlation among repeated measures of 0.5 indicated that at least 21 participants were needed to determine the variation between measurements of the number of particles per square millimeter parameter (effect size = 0.25). The Kolmogorov–Smirnov test was used to determine numerical data distribution. Repeated analysis of variance (Re-ANOVA) was used to test significant differences between the measured variables over time. Sphericity was assumed (Mauchly’s sphericity test) and post-hoc tests were used to determine significance differences in pairs of values. The intra-class correlation coefficient (ICC) was determined to assess intra-observer measurements for turbidity and PAOP values. Pearson correlation was used to test correlation between the CLDEQ-8 scores and tear meniscus parameters. Statistical significance was set at *p* < 0.05.

## Results

The study included 53 women and 22 men, and the mean age was 27.01 ± 9.73, 25.49 ± 10.01, and 28.41 ± 6.38 in groups 1, 2, and 3; respectively. The age and sex distribution among the groups did not differ significantly (*p* > 0.05). The baseline participant characteristics are represented in Table 1. The TBUT values in SCLW were significantly lower than those in ASCW and NCLW (*p* < 0.05). OSDI and CLEDQ-8 scores in SCLW were significantly higher than ASCW (*p* < 0.05). Schirmer I test results in both ASCW and NCLW were significantly higher than those of symptomatic SCL wearers (*p* < 0.05). TBUT, OSDI, and Schirmer I scores in asymptomatic SCL wearers and non-CL wearers were not significantly different (*p* > 0.05). Best-corrected visual acuity in all groups was ≤ 0.0 LogMAR.

**Table 1:** Baseline characteristics

Variables	Group 1 (SCLW)	Group 2 (ASCW)	Group 3 (NCLW)	P
Age, years				
Mean ± SD	27.01 ± 9.73	25.49 ± 10.01	28.41 ± 6.38	0.104
Sex, n				
Female	18	19	16	0.202
Male	7	6	9	
OSDI score				
Mean ± SD	16.42 ± 3.94	7.58 ± 3.71	6.01 ± 3.13	< 0.001*
TBUT, s				
Mean ± SD	4.73 ± 3.71	8.69 ± 3.86	9.38 ± 2.42	< 0.001*
Schirmer test I, mm				
Mean ± SD	7.78 ± 4.50	9.69 ± 3.72	10.92 ± 4.01	0.02*
Staining score (Oxford) (0–5)				
Mean ± SD	1.45 ± 1.06	0.37 ± 0.45	0.23 ± 0.39	0.004*
CLDEQ-8	14.48 ± 2.02	8.02 ± 2.9	NA	**0.007**†

Bold, significant differences, *OSDI* Ocular surface disease index, *TBUT* Tear break-up time, *CLDEQ* Contact Lens Dry Eye Questionnaire, *NA* not applied, *: One-Way ANOVA test, †: Independent- Samples T test, *SCLW* Symptomatic soft contact lens (SCL) wearers, *ASCW* Asymptomatic SCL wearers, *NCWL* Healthy non-CL wearers

**Fig. 2:**
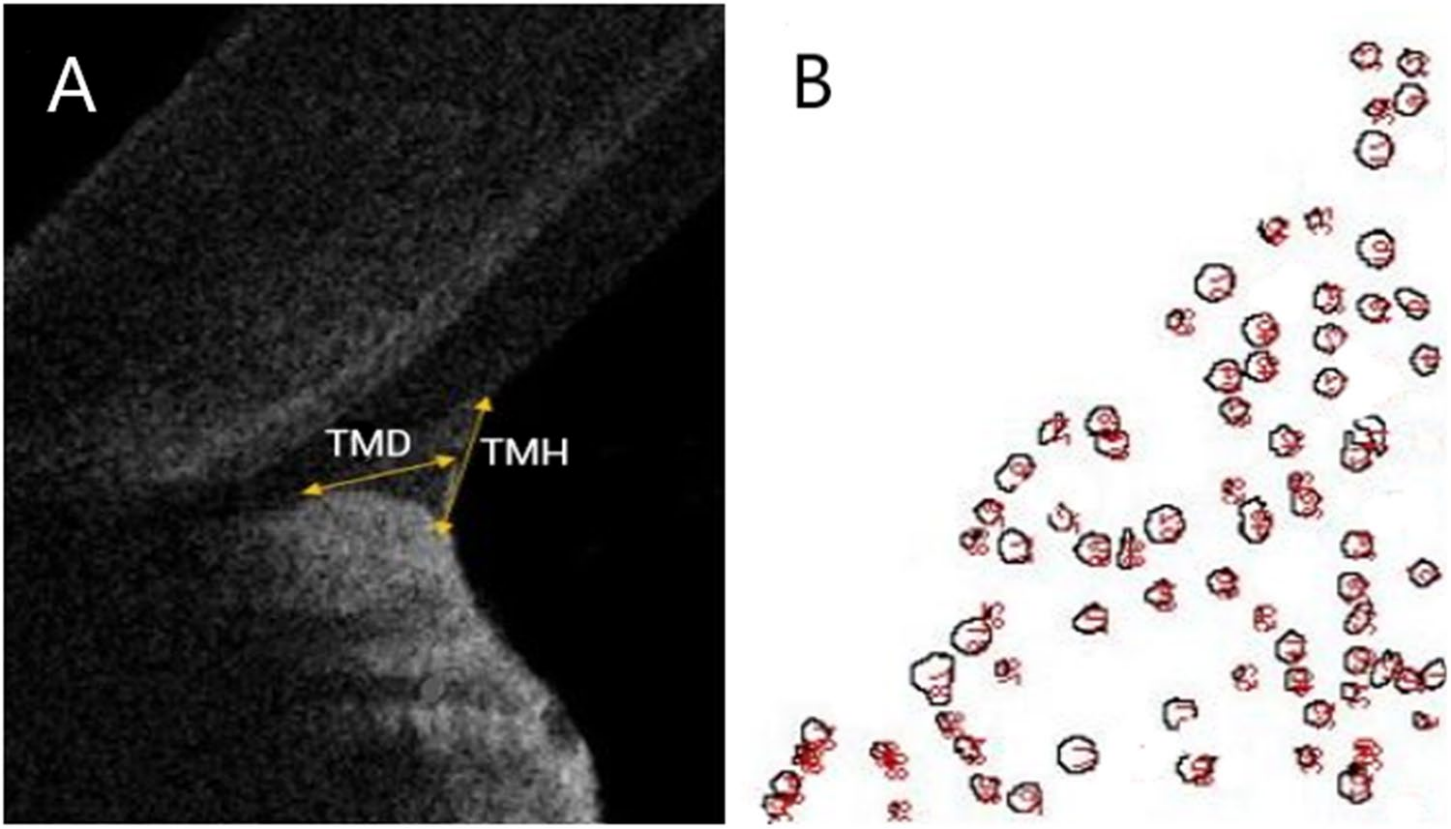
Image of measurement of tear meniscus height (TMH) and tear meniscus depth (TMD) with using optical coherence tomography in a participant with contact lens (**A**), an image taken from the ImageJ software system, which includes the particle analysis of tear meniscus (**B**)

The TMH and TMD baseline measurements in SCLW were lower than those in the other groups, but these differences were not statistically significant (*p* > 0.05). The tear meniscus turbidity and PAOP measurements at baseline in SCLW were statistically significantly higher than those in the other groups (*p* < 0.05). Although all measured tear meniscus parameters including, TMH, TMD, turbidity, and PAOP increased immediately after SCL insertion in CL wearers, only increases in TMH and TMD values were statistically significant (*p* < 0.05) (Fig. 2).

**Fig. 3:**
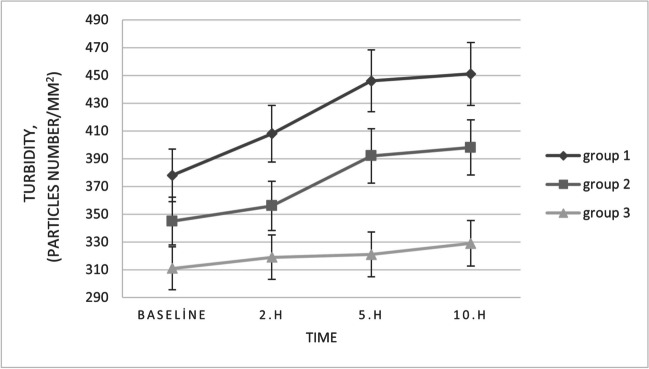
The graphs showing the changes in tear meniscus turbidity at 2, 5, and 10 h with means and standard deviations

By two hours after SCL insertion, TMH and TMD measurements had significantly decreased (Table 2) compared to measurements obtained immediately after SCL insertion (*p* < 0.05). There were no significant differences in all tear meniscus parameters between measurements at baseline and the second hour after SCL insertion (*p* > 0.05). As in the baseline measurements, turbidity and PAOP measurements in SCLW were statistically significantly higher than those in ASCW and NCLW at two hours of SCL wear (*p* < 0.05 for two comparisons) (Fig. 3).

**Table 2:**
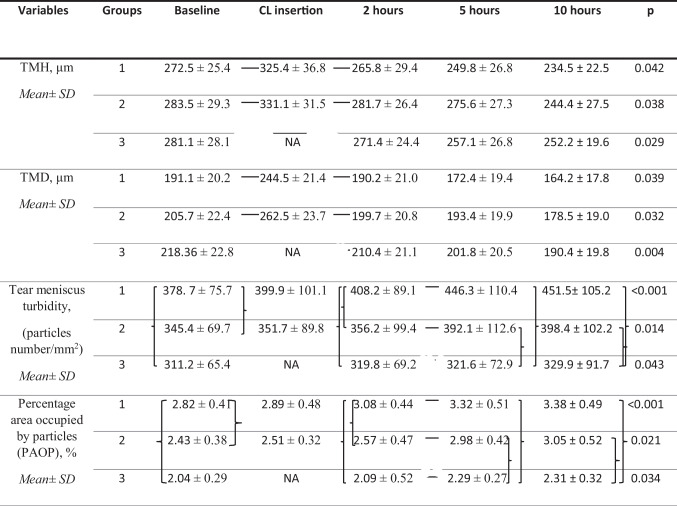
The changes in the tear meniscus parameters over time

*TMH* Tear meniscus height, *TMD* Tear meniscus depth, The lines() and brackets ({ }) indicate significant differences (*p* < 0.05). *NA* not applied, Group 1(SCLW: Symptomatic soft contact lens (SCL) wearers), Group 2 (*ASCW* Asymptomatic SCL wearers), Group 3 (*NCWL* Healthy non-CL wearers)

TMH and TMD decreased, and turbidity and PAOP increased in SCWL and ASCW at five hours of SCL wear when compared to measurements obtained after two hours of SCL wear, while the only increases in turbidity and PAOP measurements were statistically significant (*p* < 0.05). The turbidity and PAOP measurements in SCLW and ASCW at five hours of SCL wear were statistically significantly higher than the measurements obtained in the baseline visit and after two hours of SCL wear. Group 1 had significantly lower TMH and TMD measurements in the fifth hour when compared to baseline measurements (*p* < 0.05). Additionally, turbidity and PAOP measurements in SCL wearers (SCLW and ASCW) were significantly higher than those in NCLW at five hours of SCL wear (*p* < 0.05).

**Fig. 4:**
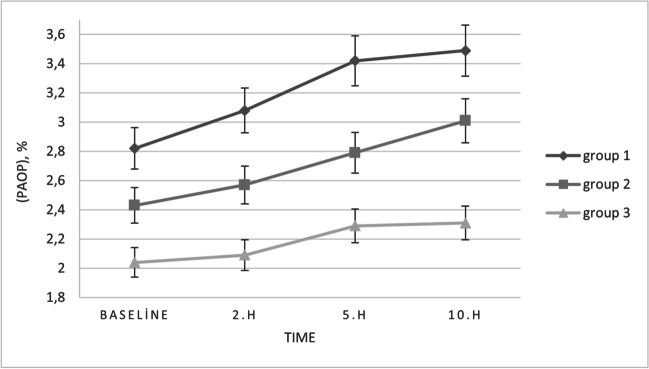
The graphs showing the changes in percentage area occupied by particles (PAOP) in the tear meniscus at 2, 5, and 10 h with means and standard deviations

TMH and TMD measurements in all groups at 10 h of SCL wear were the lowest among the values that were recorded in each visit. Nonetheless, TMH and TMD measurements in SCLW and ASCW were significantly lower after 10 h of SCL wear than only the values recorded during the baseline visit (*p* < 0.05). Group 1 had significantly higher turbidity and PAOP results than the other groups at 10 h of SCL wear (*p* < 0.001 for two comparisons). As in the measurements after five hours of SCL wear, turbidity and PAOP in SCL wearers (SCLW and ASCW) were significantly higher than in non-CL wearers after 10 h of SCL insertion (*p* < 0.05) and there were no statistically significant differences in turbidity and PAOP measurements between fifth- and tenth-hour records of all groups. At the end of the tenth hour, tear meniscus volume in the NCLW group had significantly increased (*p* < 0.05), turbidity and PAOP values showed an increase, however these changes were not statistically significant compared to the baseline visit (*p* > 0.05) (Fig. 4).

The ICC values for intra-observer agreement (95% CI) was calculated as 0.91 (0.90- 0.92), and 0.89 (0.88- 0.90) (*p* < 0.001) for turbidity and PAOP measurements, sincerely. Turbidity and PAOP had a positive correlation with each other in all the participants' OCT examinations (*r* = 0.436, *p* < 0.05). The CLDEQ-8 results were positively correlated with turbidity and PAOP measurements obtained at five and ten hours of SCL wear in all symptomatic and non-symptomatic SCL wearers (*r* = 0.305, *p* < 0.05).

## Discussion

**Fig. 5:**
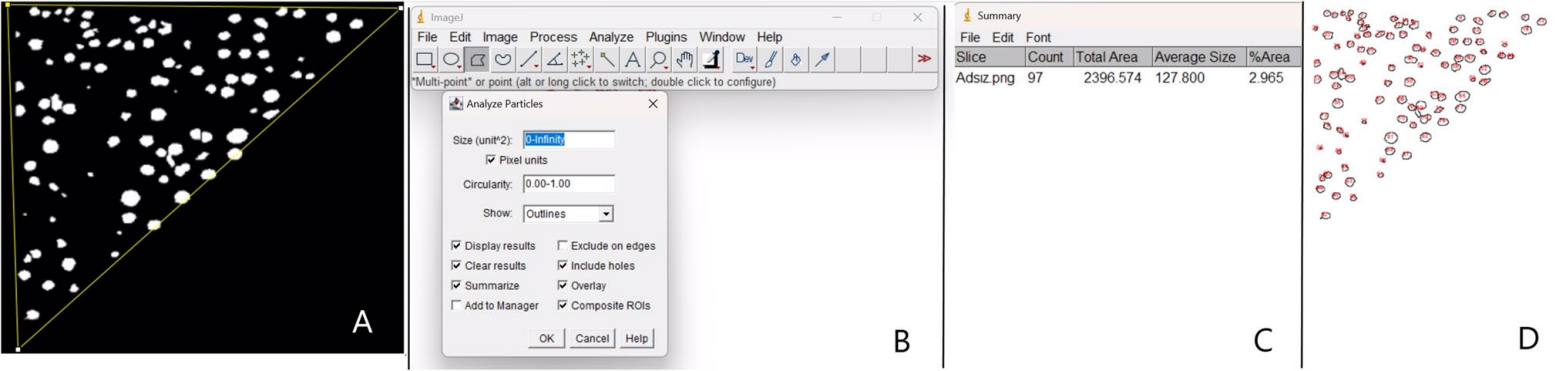
Utilizing the freehand selection tool to carefully outline the meniscus area and converting the selected area into black-and-white image in the ImageJ software (**A**), particles analysis for the obtained image using the ‘Analyze particles’ window (**B**), following the particle analysis, the "Analyze Particles" window will display the results, including the total area of the analyzed particles, the number of detected particles, and the mean particle area (PAOP) (**C**), counting the number of particles identified within the selected meniscus area (**D**)

Tear meniscus is an important indicator of sufficient tear volume [29]. In addition to sufficiency of tear volume, an optimal quality of tears is necessary for a healthy tear film turnover [29]. In previous studies, TMH and TMD parameters have been analyzed with various methos [14, 20, 30]. In this study, tear meniscus parameters including turbidity, PAOP, TMH and TMD non-invasively in SCL wearers (symptomatic and non-symptomatic) and healthy participants were evaluated using SD-OCT and ImageJ software. Wikaninburg et al.'s work demonstrated the potential of ImageJ by calculating turbidity levels in water pollution evaluation, particularly for quick screening or resource-limited settings [31]. They showed that to assess the potential for turbidity level prediction based on the calculation of the number and surface area of suspended particles with ImageJ [31]. Additionally, Bessell- Browne et al. did indeed find that turbidity in water is mainly affected by suspended sediments and sediment particle size [32]. Carracedo et al. used the ImageJ software system to evaluate the tear meniscus turbidity changes after lubricating drops, and post-lens aqueous fluid turbidity changes in scleral contact lens users [24, 27] (Fig. 5).

In this study, symptomatic SCL wearers had significantly lower baseline TBUT, and Schirmer I scores and their OSDI scores were significantly higher than the other groups. However, TMH, and TMD measurements in SCLW were not significantly lower than in the other groups, the turbidity and PAOP results, calculated using ImageJ software, were significantly higher in SCLW’s baseline measurements. The tear meniscus turbidity was calculated as the number of particles per square millimeter, and PAOP was the ratio of total area occupied by particles to tear meniscus volume. When considering the increased turbidity and PAOP measurements in symptomatic SCL wearers, the particle number in the tear meniscus must have increased. According to previous studies, inflammatory cells, and tear meniscus osmolarity increase in CL users and especially in symptomatic wearers [33–35]. Additionally, incidence of meibomian gland dysfunction increases in CL users [33], and this condition can be another reason why the turbidity and PAOP results are high even in the baseline OCT measurements in the symptomatic SCL wearers.

The highest TMD and TMH measurements were obtained immediately after SCL insertion. The increased tear meniscus volume can be explained by the extra fluid brought into the eyes with the lens and the reflex tearing aggravated by CL insertion [23]. The turbidity and PAOP also increased with the insertion of SCLs, while there were not any significant differences with baseline measurements in CL wearers. Abusharha et al. reported that the osmolarity of basal and reflex tears was not statistically significantly different [36]. Additionally, the usage of daily disposable SCL diminished the negative effects of contact lens care solution on the turbidity and PAOP results in the present study.

At two hours of SCL wear, the all-tear meniscus parameters recovered and approached the baseline measurements (Table 2), and only the symptomatic participants had statistically significantly higher turbidity and PAOP results than the other groups. Being a CL wearer is not enough to explain the increase in the turbidity at two hours because the turbidity and PAOP measurements were not significantly different between non-symptomatic SCL wearers and healthy non-CL wearers. Although Wang et al. showed that tear meniscus volume after CL insertion returned to the baseline values and remained that way for at least four hours in adapted wearers [23], they did not evaluate the turbidity and PAOP parameters. In this study, although turbidity and PAOP were significantly higher in all SCL wearers (SCLW and ASCW) than those in healthy non-CL wearers at the fifth hour, TMH and TMD only in SCLW were significantly lower than those in NCLW at the same visit.

The last OCT examinations were performed at 10 h of SCL wear. The all-tear meniscus parameters significantly changed compared to the baseline in CL wearers. Additionally, turbidity and PAOP values in SCLW and ASCW were significantly higher than those in NCLW in the tenth hour with SCL. The diminished mucin and lipid layer on the lens surface with a long period of SCL wear increased tear evaporation, and this condition may contribute to reduced tear meniscus volumes and increased tear osmolarity [37]. It hypothesized that increased inflammatory cells, decreased tear meniscus volume, and tear hyperosmolarity would lead to higher tear meniscus turbidity and PAOP values in both symptomatic and non-symptomatic SCL wearers in the present study. Moreover, the precise identification of the cells responsible for the elevated number and density of particles within CL wearers' tear meniscus remains elusive. The presence of increased epithelial shedding, potentially in response to the toxic effects of contact lens solutions in addition to the previously mentioned inflammatory mechanisms, may contribute to this phenomenon [38]. Desquamated epithelial cells or cells that naturally shed from the ocular surface, along with tear film proteins and lipids, can adhere to CLs, thereby contributing to deposit formation [38].

Even in healthy people**,** tear film stability and tear break-up time decrease, while evaporation of tear fluid, tear osmolarity, inflammatory molecules in tears, and ocular discomfort increase throughout the day, especially in the evening hours [39–42]. Considering that turbidity is the ratio of the particles in the meniscus to the total volume, increased evaporation, decreased tear meniscus volume, and increased tear osmolarity may explain the possible increase in particle ratio in tear meniscus over time, leading to increased turbidity. Increased osmolarity and inflammation may trigger not only the increased particle ratio but also the aggregation of these particles, which may increase the area occupied by the particles in the meniscus. In this study, as Ayaki et al. stated, TMH and TMD decreased in healthy participants in the later hours [43], while the increases in turbidity and PAOP values were not significant.

In the case of healthy participants**,** there was a trend toward a decrease in tear meniscus volume and an increase in turbidity and PAOP after baseline measurement**.** The changes in tear meniscus volume reached statistical significance by the tenth hour**.** However, in symptomatic contact lens users, we observed a significant decrease in TMD and TMH starting as early as the fifth hour of SCL wear. Furthermore, the turbidity and PAOP values in the SCLW group were notably higher even during baseline measurements compared to participants in the ASCW and NCLW groups.

This study has some limitations. First, the CL wearers were not evaluated according to the type of CL they used before the study. Only daily disposable CLs were used in the study, and no comparison was made with reusable CL. Second, the tear meniscus parameters were evaluated in a relatively short time period. New studies with contact lenses that have different materials or reusable properties and over a longer period are required to evaluate the potential effects of CL wear on tear meniscus parameters.

In summary, TMH and TMD decrease in all participants at tenth hour, while tear meniscus turbidity and PAOP in SCL wearers increase at fifth hour. Despite these findings, symptomatic SCL users exhibited a significant decrease in TMH and TMD, even at fifth hours. Tear meniscus volume in asymptomatic SCL users and healthy participants did not show significant changes until 10 h, while PAOP and turbidity showed a significant increase in all SCL users at the 5th hour. It is known that contact lens users experience increased ocular discomfort starting at the second hour, however PAOP and turbidity did not show significant changes at the 2nd hour with SCL use. This situation may be explained by the fact that SCL wearers in this study were divided into two groups according to their dry eye symptoms, and the symptomatic participants had higher turbidity and PAOP values than healthy individuals since the baseline measurements. Even if the tear meniscus volume was not significantly different between the groups at baseline and at two hours of SCL wear, turbidity and PAOP were statistically significantly higher in symptomatic SCL wears. At five hours of SCL wear, even asymptomatic SCL wearers had significantly higher turbidity and PAOP measurements than healthy non-SCL wearers. While contact lenses narrow the tear meniscus, they do not have a significant effect on the geometry of the meniscus anatomically [44, 45]. Nevertheless, as the number of hours spent wearing contact lenses increases throughout the day, this can lead to an increase in the factors that play a role in the increase of tear film turbidity and PAOP. Turbidity and PAOP may be measures of how well the tear film and meniscus are functioning and they maybe one of the indicators of dry eye in SCL wearers. Lastly, SD-OCT can be helpful in the non-invasive evaluation of tear meniscus in CL wearers.

## Data Availability

The data used to support the findings of this study are included in this article.
